# Eosinophils in Autoimmune Diseases

**DOI:** 10.3389/fimmu.2017.00484

**Published:** 2017-04-27

**Authors:** Nicola L. Diny, Noel R. Rose, Daniela Čiháková

**Affiliations:** ^1^W. Harry Feinstone Department of Molecular Microbiology and Immunology, Johns Hopkins University Bloomberg School of Public Health, Baltimore, MD, USA; ^2^Department of Pathology, Brigham and Women’s Hospital, Harvard Medical School, Boston, MA, USA; ^3^Department of Pathology, Johns Hopkins University School of Medicine, Baltimore, MD, USA

**Keywords:** innate immune system, autoimmune diseases, eosinophilia, bullous pemphigoid, neuromyelitis optica, eosinophilic granulomatosis with polyangiitis, myocarditis, inflammatory bowel disease

## Abstract

Eosinophils are multifunctional granulocytes that contribute to initiation and modulation of inflammation. Their role in asthma and parasitic infections has long been recognized. Growing evidence now reveals a role for eosinophils in autoimmune diseases. In this review, we summarize the function of eosinophils in inflammatory bowel diseases, neuromyelitis optica, bullous pemphigoid, autoimmune myocarditis, primary biliary cirrhosis, eosinophilic granulomatosis with polyangiitis, and other autoimmune diseases. Clinical studies, eosinophil-targeted therapies, and experimental models have contributed to our understanding of the regulation and function of eosinophils in these diseases. By examining the role of eosinophils in autoimmune diseases of different organs, we can identify common pathogenic mechanisms. These include degranulation of cytotoxic granule proteins, induction of antibody-dependent cell-mediated cytotoxicity, release of proteases degrading extracellular matrix, immune modulation through cytokines, antigen presentation, and prothrombotic functions. The association of eosinophilic diseases with autoimmune diseases is also examined, showing a possible increase in autoimmune diseases in patients with eosinophilic esophagitis, hypereosinophilic syndrome, and non-allergic asthma. Finally, we summarize key future research needs.

## Introduction

The cells of the innate immune system can contribute to autoimmune diseases. Activation of innate immune cells by pathogen-associated molecular patterns and antigen presentation by dendritic cells can result in priming of autoreactive T and B cells and set off an adaptive immune response against self-antigens (1–3). Possible roles for innate immune cells exist not only in the initiation stage of autoimmune diseases but also in the modulation and propagation of inflammation and tissue destruction. Such roles have been proposed for neutrophils (4), natural killer cells (5, 6), macrophages (7), dendritic cells (8, 9), innate lymphoid cells (10), and mast cells (11). Eosinophils have been recognized as a part of the inflammatory infiltrate in several organ-specific autoimmune diseases, but their potential role in autoimmune diseases has not been addressed comprehensively. The aim of this review is to synthesize the role of eosinophils in different autoimmune diseases and explore potential unifying effector mechanisms. We also address the association of autoimmune diseases with eosinophil-associated disease like asthma and eosinophilic esophagitis.

## Eosinophils

### Eosinophil Biology

Eosinophils are granulocytes that develop in the bone marrow in response to IL-5, with a minor role for IL-3, granulocyte-macrophage colony-stimulating factor (GM-CSF), and IL-33 (12–15). IL-5 also mediates the release of mature eosinophils into the bloodstream from where they migrate into tissues (16). In healthy individuals, eosinophils are found in the bone marrow, blood, spleen, thymus, gastrointestinal tract, and uterus (17). Under pathological conditions, eosinophils can infiltrate other tissues as well. Eosinophils are usually enumerated in the blood because tissue eosinophils are hard to measure. Eosinophil counts over 450–500 cells/μl blood are considered mild eosinophilia and counts over 1,500 cells/μl are characterized as hypereosinophilia (18).

**Figure 1:**
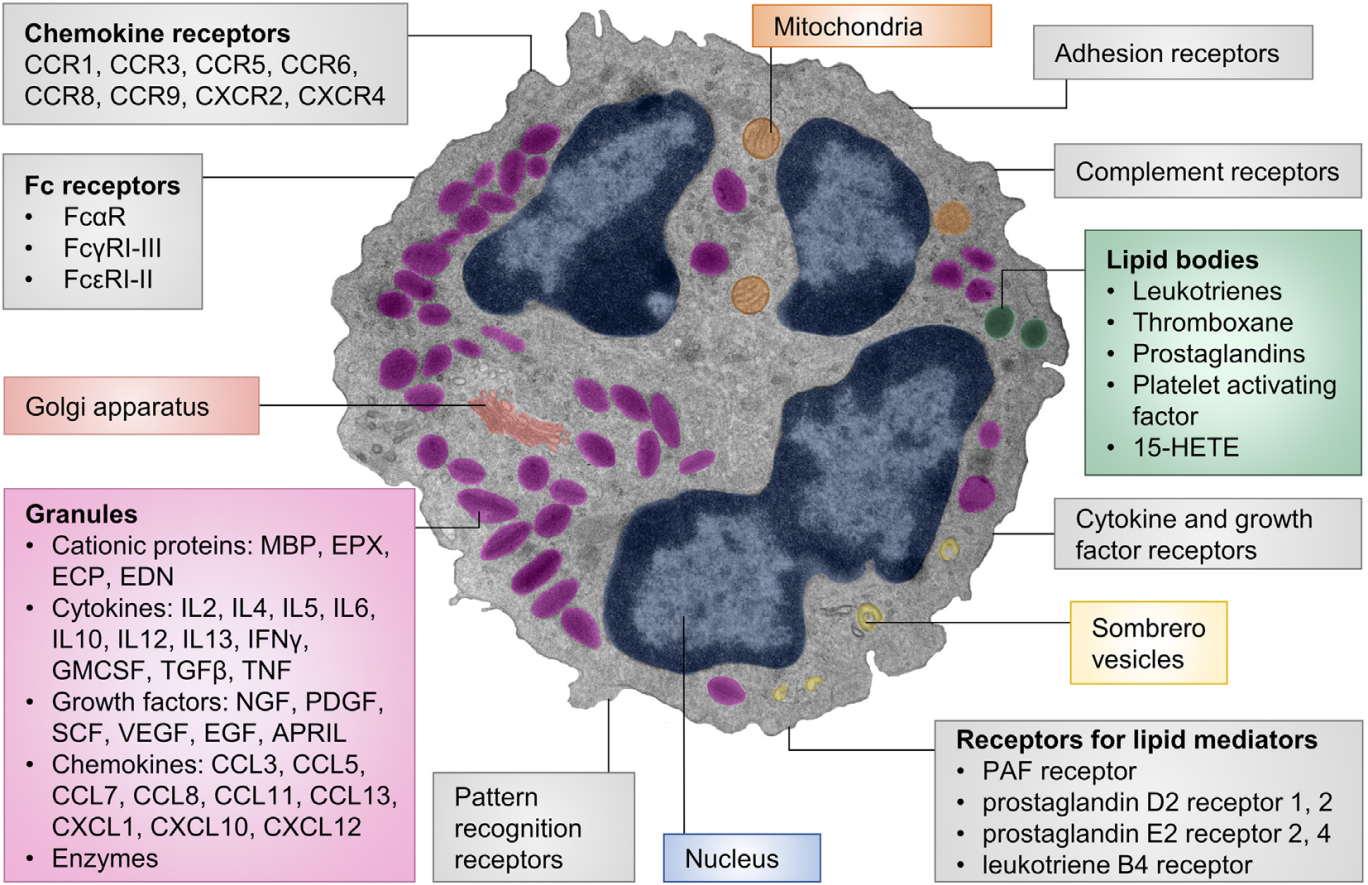
**Cellular structure, receptors, and mediators of eosinophils**. The pseudocolored composite electron micrograph of an eosinophil highlights cellular structures. Characteristic features of eosinophils include the multilobed nucleus, specific eosinophil granules, lipid bodies, and sombrero vesicles. Eosinophil granules contain cationic proteins, cytokines, growth factors, chemokines, and enzymes. The granule contents can be released upon stimulation. Lipid bodies are the place of synthesis for numerous lipid mediators. Granule contents can be released through sombrero vesicles. Eosinophils carry numerous cell surface receptors including chemokine receptors, Fc receptors, pattern recognition receptors, receptors for lipid mediators, cytokine receptors, complement receptors, and adhesion receptors. Abbreviations: 15-HETE, 15-hydroxyeicosatetraenoic acid; APRIL, a proliferation-inducing ligand; CCL, CC-chemokine ligand; CCR, CC-chemokine receptor; CXCL, CXC-chemokine ligand; CXCR, CXC-chemokine receptor; ECP, eosinophil cationic protein; EDN, eosinophil-derived neurotoxin; EGF, epidermal growth factor; EPX, eosinophil peroxidase; GMCSF, granulocyte-macrophage colony-stimulating factor; IFN, interferon; MBP, major basic protein; NGF, nerve growth factor; PDGF, platelet-derived growth factor; PAF, platelet-activating factor; SCF, stem cell factor; TGF, transforming growth factor; TNF, tumor necrosis factor; VEGF, vascular endothelial growth factor. The electron micrograph was generously provided by Dr. Isabelle Coppens, Johns Hopkins University, Baltimore, MD, USA.

The main chemotaxins for eosinophils are eotaxins, which homeostatically recruit eosinophils to the gastrointestinal tract, thymus, and uterus (19–21) and to other organs in disease states (22–24). Humans express three functional eotaxins (CCL11, CCL24, and CCL26), whereas mice only express two (CCL11 and CCL24) (25–28). The eotaxin receptor, CCR3, is highly expressed on eosinophils and to a low level on human basophils, mast cells, and Th2 cells (29, 30). Other eosinophil chemoattractants include CCL5 and lipid mediators such as leukotriene B4 and prostaglandin D2, although these factors are not specific for eosinophils (31) (Figure 1).

A unique characteristic of eosinophils are their specific (also termed secondary or secretory) granules. These are secretory vesicles with an electron-dense core and an electron-lucent matrix. Eosinophil granules contain four major granule proteins and numerous cytokines, chemokines, and growth factors (31) (Figure 1). Cytotoxic effects to host tissues and pathogens have been demonstrated for all major granule proteins: eosinophil cationic protein (ECP), eosinophil-derived neurotoxin (EDN), eosinophil peroxidase (EPX), and major basic protein (MBP) (32). MBP can disrupt the cell membrane and is therefore highly cytotoxic to mammalian cells, helminths, and bacteria (33–35). Other effects of MBP include altering smooth muscle contraction, inducing mast cell and basophil degranulation, provoking acetylcholine release from peripheral nerves, and promoting nerve cell survival (36–39). The granule proteins ECP and EDN are ribonucleases (13, 40) with neurotoxic and strong antiviral activities (41, 42) and immune modulatory functions (43). EPX generates reactive oxygen species that are directed extracellularly (44). These products have cytotoxic, prothrombotic, and pro-inflammatory effects (44–46). Granule contents are generally preformed in eosinophils and released upon stimulation. Piecemeal degranulation is the most common process by which eosinophils release their granule contents (47–49). Specific granule factors, rather than the entire granule, are released in response to an activating signal. This leaves the eosinophil intact and able to respond to subsequent stimulation.

### Possible Eosinophil Effector Functions in Autoimmune Diseases

Eosinophils are extremely versatile effector cells that damage tissues or modulate the activity of other immune and stromal cells. One could envision many of these effector functions playing a role in the context of autoimmune diseases as well (Box 1). Damage of tissues and cells is a feature of many organ-specific autoimmune diseases. Eosinophils are well known for their strong cytotoxic properties, mediated mostly through granule proteins. This could contribute to organ destruction in autoimmune inflammation.

Box 1 **Possible eosinophil effector functions in autoimmune diseases.**

- Damage of tissues by cytotoxic granule proteins
- Antibody-dependent cellular cytotoxicity
- Activation of tissue remodeling and fibrosis
- Antigen presentation
- Modulation of the adaptive immune response
- Promotion of B cell responses
- Induction of tissue repair processes.

The ability of eosinophils to bind antibodies and consequently degranulate and kill cells links the adaptive autoimmune response to eosinophil effector functions. Eosinophils express complement receptors (50) and Fc receptors (FcαR, FcγRI–III, and FcεRI–II) either constitutively or under inflammatory conditions (51–54). As a result, they are capable of antibody-dependent cellular cytotoxicity (ADCC) to parasites and mammalian targets (55–57). In autoimmune diseases, eosinophils may kill host cells bound by autoantibodies.

Eosinophils also interact with stromal cells. Actively degranulating eosinophils are frequently found in areas of fibrogenesis, suggesting a potential profibrotic role (58–61). Granule proteins and eosinophil-derived transforming growth factor (TGF)β1 were demonstrated to affect tissue remodeling and fibrosis. Eosinophils can promote fibroblast proliferation (62–64), proteoglycan accumulation (65), matrix metalloproteinase and TGFβ expression, and extracellular matrix protein synthesis (66). These profibrotic functions of eosinophils may add to tissue dysfunction in autoimmune diseases. In chronic inflammatory conditions, eosinophils preferentially locate to nerves (67–70). This interaction results in activation of eosinophils (71–73), nerve damage (74, 75), altered nerve growth (76, 77), and neuropeptide release (78). Contact between eosinophils and nerves has functional consequences. For example, it is one of the causes of airway hypersensitivity in asthma (38, 79).

Eosinophils can form extracellular DNA traps by quickly releasing mitochondrial DNA and granule proteins (80). These structures bind and kill pathogens and contribute to tissue injury in inflammatory conditions (81). DNA extracellular traps have been described in allergic asthma (82), drug hypersensitivity reactions, and allergic contact dermatitis (83). Eosinophils may initiate or perpetuate inflammation by releasing cytokines and chemokines and by interacting with other innate immune cells. For example, eosinophils release MBP, IL-9, stem cell factor, or nerve growth factor, which affect mast cell maturation, survival, and histamine release (84–87).

Eosinophils can also influence the adaptive immune response. They are capable antigen-presenting cells that upregulate MHCII and costimulatory molecules in the context of parasitic infection or allergic asthma (88–90). Moreover, eosinophils migrate to draining lymph nodes (91), and *in vitro* experiments have demonstrated their ability to present antigen to and activate T cells (92–94). Eosinophils may contribute to the initiation of autoimmune responses by presenting antigen to and activating T cells.

Eosinophil granules contain numerous cytokines such as IL-4, IL-13, IL-25, TGFβ, IL-10, or IDO (31), which suggests an ability to affect T cell differentiation. Eosinophils were shown to suppress Th1/Th17 differentiation (95) or activate Th2 responses in draining lymph nodes (96). In addition, they modulate dendritic cell activity, thereby indirectly affecting polarization of naïve T cells into Th2 cells (97). Eosinophils also shape the humoral immune response. In the bone marrow, eosinophils stimulate plasma cell survival by producing IL-6 and a proliferation-inducing ligand (98), and in the intestine, they promote class-switching to IgA (99, 100). These properties enable eosinophils to shape the adaptive immune response in autoimmune diseases.

Eosinophils may also fulfill immune regulatory and protective functions. Eosinophil-derived mediators like TGFβ and TGFα (101), platelet-derived growth factor (102), vascular endothelial growth factor (103), and fibroblast growth factor (104) can all contribute to tissue repair and angiogenesis. IL-4 released from eosinophils was shown to play a role in liver (105) and muscle (106) regeneration. Whether eosinophils contribute to tissue repair or tissue damage is likely context and disease dependent.

### Identification of Eosinophils in Blood and Tissues

Numerous methods exist to identify eosinophils in blood and tissues. Blood eosinophils are routinely counted in clinical settings in differential white blood counts. Human eosinophils (and to a lesser extent mouse eosinophils) are easily identified by hematoxylin and eosin staining of histological sections due to the bright pink staining of the basic granules, which gave them the name eosinophils (107). Mouse blood eosinophils can be detected using modified Giemsa stain or by flow cytometry. Their characteristic forward scatter-side scatter profile in flow cytometry allows for approximation of eosinophils in blood even without specific antibody staining. Antibodies that can be used to stain blood eosinophils target Siglec-F (mouse) or CCR3 (mouse and human) (108, 109). Eosinophils are also positive for unspecific receptors such as CD11b and (at low/intermediate levels) Ly6G. Staining with several antibodies is often required for identification and characterization of eosinophils from tissues, as none of them are absolutely specific for eosinophils (110). Tissue eosinophils can also be detected using immunohistochemistry or immunofluorescence staining with antibodies against MBP or EPX (111). Electron microscopy is another method used to distinguish eosinophils from other cells based on the morphology of eosinophil granules. Moreover, with this technique, the extent and mechanism of degranulation of eosinophils can be determined (112).

## Role of Eosinophils in Autoimmune Diseases

### Bullous Pemphigoid

Bullous pemphigoid is a blistering disease of the skin with a well-established autoimmune etiology (113). Autoantibodies bind to hemidesmosomal proteins BP180 and BP230 at the dermal–epidermal junction and other extracellular matrix proteins (114–119). Hemidesmosomes are part of the complexes that anchor the cytoskeleton of basal keratinocytes to the dermis (120). Autoantibody binding triggers complement activation, recruitment of immune cells, and release of proteases. This results in tissue damage and blistering (121–124). Neutrophils and eosinophils infiltrate the dermal–epidermal junction and, together with mast cells, are thought to play a key role in bullous pemphigoid (125–127). Increased numbers of peripheral blood eosinophils has long been recognized as a characteristic of bullous pemphigoid patients (128, 129). A positive correlation between blood eosinophil numbers and disease severity has been observed in some reports (130, 131) but not others (132). Eosinophilia in bullous pemphigoid patients is likely caused by increased levels of IL-5, which can be detected at high levels in the serum and blister fluid (133, 134). Keratinocytes in the blisters express eotaxin-1, which directs eosinophil infiltration (133, 135). Eotaxin-1 expression is positively correlated with the number of infiltrating eosinophils in blisters. Eosinophil localization to the basement membrane zone is autoantibody and complement dependent in a human cryosection model of bullous pemphigoid (127). Eosinophils from blisters release IL-6, IL-8, and IL-1β and show an activated phenotype with high CD11b expression (136). Blister eosinophils also underwent apoptosis more readily compared to eosinophils from healthy donors (136).

Several mechanisms by which eosinophils (and other granulocytes) contribute to lesion formation have been identified. Eosinophils in lesional skin were shown to degranulate, and granule proteins are deposited in blisters (125, 137). The eosinophil granule protein ECP can be readily detected in serum and blister fluid of bullous pemphigoid patients (132). However, it is not clear if granule proteins contribute to tissue damage. Eosinophils and neutrophils have been shown to release proteases, matrix metalloproteinase 9 (MMP9), and neutrophil elastase, in lesional biopsies and blister fluid. These proteases can degrade extracellular matrix proteins and BP180, which contributes to dermal–epidermal separation and blister formation (138–140). Blister formation also depends on autoantibodies (141), which are of the IgG1, IgG4, and IgE subtype (142, 143). Recently, eosinophils from bullous pemphigoid patients were shown to express the high-affinity IgE receptor FcεRIα (144), which may trigger eosinophil activation by IgE autoantibodies. Additional evidence for a pathogenic role for eosinophils comes from a case report of a patient with hypereosinophilic syndrome and bullous pemphigoid who was treated with imatinib (a tyrosine kinase inhibitor). In response to imatinib, both conditions resolved and his eosinophil count normalized (145).

Taken together, there is strong evidence from patient studies, *in vitro* experiments, and animal models for a pathogenic role of eosinophils in bullous pemphigoid. In addition to the mouse model of passive antibody transfer, which reproduces blister formation but not eosinophil infiltration (146), a new model with genetically modified mice has been established (147). Mice with a deletion in the BP180 (collagen XVII) gene spontaneously develop eosinophilia, blister formation, itch, and eosinophil infiltration into the skin lesions. This new model could be used to test for the requirement and pathologic role of eosinophils (and eosinophil products) in future studies. Novel eosinophil-specific drugs may also help to clarify the role of eosinophils in bullous pemphigoid. Trials of bertilimumab, an anti-eotaxin-1 antibody, and mepolizumab, an anti-IL-5 antibody, are currently ongoing (http://ClinicalTrials.gov identifiers: NCT02226146 and NCT01705795).

### Inflammatory Bowel Diseases

The etiology of the inflammatory bowel diseases, Crohn’s disease and ulcerative colitis, is not fully understood. Evidence for the involvement of autoimmune processes exists for both (148, 149). Both diseases are associated with other autoimmune diseases, characterized by lymphocytic infiltration, and respond to corticosteroid treatment. Patients with ulcerative colitis carry autoantibodies against colonic epithelial cells and often perinuclear antineutrophilic cytoplasmic (ANCA) antibodies. Specific autoantibodies have not been found in Crohn’s disease patients. The evidence for autoimmunity is stronger in ulcerative colitis than in Crohn’s disease. Here, we will discuss the role of eosinophils in both diseases with a focus on ulcerative colitis.

Eosinophils have long been recognized as a prominent feature of the infiltrate in inflammatory bowel diseases (150–154). Eosinophil numbers in the colon are substantially increased in inflammatory bowel disease patients and display an activated phenotype (154–157). The number of infiltrating eosinophils is positively correlated with disease severity in ulcerative colitis and Crohn’s disease (23, 158–161). In mouse models, the absence of eosinophils dramatically reduces disease severity. In the model of DSS-induced colitis, two different strains of eosinophil-deficient mice were protected compared to controls (23, 162). Depletion of eosinophils in a model of colitis due to *Helicobacter hepaticus* infection also reduced disease severity (163). Similarly, in a model of TNBS-induced colitis, eosinophil-deficient mice fared better, while hypereosinophilic mice developed more severe disease (164).

Eosinophil migration into the colon mucosa occurs in response to eotaxins. Patients with inflammatory bowel diseases have elevated serum eotaxin-1 levels (158, 165, 166), which correlates positively with disease activity (158, 165). Tissue expression of eotaxin-1, and to a lesser extent eotaxin-2, is increased in ulcerative colitis patients and positively correlated with the number of infiltrating eosinophils and histopathologic disease severity (23, 158). Another study found increased expression of all three eotaxins and IL-5 in ulcerative colitis, but only eotaxin-1 correlated with eosinophil numbers (167). The relative significance of eotaxin-2 and -3 is less clear. Eotaxin-3 was found to be increased in active lesions in ulcerative colitis and to a lesser extent in Crohn’s disease (168). Gene polymorphisms in eotaxin-2 are associated with ulcerative colitis (169). This suggests that all eotaxins may contribute to eosinophil trafficking. The cellular source of eotaxin-1 was identified as CD68^+^ macrophages and epithelial cells (23) or as CD14^+^ mononuclear cells (167). Colonic myofibroblasts express eotaxin-3, which is increased in response to IL-4 and IL-13 (168). In the mouse model of DSS-induced colitis, expression of eotaxin-1 and -2 in the colon is increased and deficiency of eotaxin-1, but not eotaxin-2, decreases eosinophil infiltration. This demonstrates that eotaxin-1 is the major chemoattractant for eosinophils in experimental colitis (23, 170). In this mouse model, macrophages are the major eotaxin-1-producing cell type (23, 171). The increased expression of eotaxins, particularly eotaxin-1, in inflammatory bowel diseases shows that eosinophils are specifically recruited to the site of inflammation.

Electron microscopy and immunohistochemistry of colonic biopsies show degranulation of eosinophils (157, 172, 173). Eosinophil granule proteins are also found in the feces (156, 174), gut perfusates (175), and gut lavage fluids (176) of patients with ulcerative colitis and Crohn’s disease. Eosinophil granule proteins in serum or intestine are positively correlated with disease activity in ulcerative colitis (23, 156, 161, 177, 178). Polymorphisms in the genes of ECP and EPX are associated with inflammatory bowel diseases (179). These findings suggest a pathogenic role of eosinophil granule proteins in inflammatory bowel diseases. In one study, however, eosinophil activation was observed during the remission phase (180). EPX is pathogenic in mouse models of DSS- and *H. hepaticus*-induced colitis. Genetic deficiency or inhibition of EPX reduced disease severity (163, 170).

Several pathogenic functions of eosinophils have been suggested in recent years. Eosinophils were found to increase mucosal barrier permeability in ulcerative colitis by releasing MBP (181) or corticotropin-releasing factor (182). IL-22 is increased in patients with ulcerative colitis or Crohn’s disease (183, 184), and animal studies showed that it is crucial to restore epithelial homeostasis (185). IL-22 induces antimicrobial peptides, mucus production, and epithelial tight junctions (186). Recently, eosinophils were identified as the main source of IL-22-binding protein (IL-22BP), inhibiting the protective actions of IL-22 in DSS-induced experimental colitis and in patients with inflammatory bowel disease (187). In another study, eosinophils were found to localize to nerves in the colonic mucosa in ulcerative colitis and Crohn’s disease (70). Th17 responses have been implicated in inflammatory bowel diseases (188). A possible link between the downstream effector of Th17 responses, GM-CSF, and eosinophils was found recently. GM-CSF enhances eosinophilopoiesis, induces cytokine secretion from eosinophils, and promotes eosinophil survival (163, 189).

In summary, tissue eosinophils are increased in patients with inflammatory bowel diseases, are associated with disease severity, and are specifically recruited through eotaxin-1. Eosinophils likely contribute to the disease process by releasing granule proteins (EPX) or other mediators that affect the intestinal barrier. Thus, there is a strong evidence for a pathogenic role of eosinophils in inflammatory bowel diseases, particularly in ulcerative colitis.

### Eosinophilic Granulomatosis with Polyangiitis (EGPA)

Eosinophilic granulomatosis with polyangiitis was first described by Churg and Strauss in 1951 (190). The disease progresses through three overlapping phases: adult-onset asthma, peripheral and tissue eosinophilia, and necrotizing vasculitis with tissue infiltration of eosinophils (191–193). EGPA is an idiopathic type of small vessel vasculitis and is also part of the hypereosinophilic syndromes (194). It is associated with HLA and IL-10 polymorphisms (195). About 40% of EGPA patients have perinuclear ANCA antibodies against myeloperoxidase (MPO), resulting in the classification of EGPA as an ANCA-associated vasculitis (192, 193). The presence or absence of ANCA in EGPA may indicate two clinical subtypes with different organ involvement. ANCA-positive patients have more frequent vasculitis and glomerulonephritis, whereas ANCA-negative patients have more frequent heart and lung involvement (196, 197).

Blood and tissue eosinophilia are diagnostic criteria for EGPA, yet little is known about the pathogenic role of eosinophils in this disease (192). One reason for the absence of mechanistic data is the lack of suitable animal models. The transfer of MPO-positive human serum to mice causes vasculitis, but the eosinophilic component is missing (198). Therefore, all knowledge about the role of eosinophils in EGPA comes from patient studies. An increased eosinophil count during active disease is associated with increased Th2 cytokines IL-5 in serum (199, 200) and increased production of IL-4, IL-5, and IL-13 by T cells (201, 202). CCL17, a chemokine that recruits Th2 cells into tissues, is increased in the serum and biopsies of EGPA patients and is positively correlated with blood eosinophils and IgE (199, 203). This increase in Th2 activity likely contributes to eosinophilia.

Blood eosinophils in EGPA show an activated phenotype expressing high levels of CD69 and CD11b (200, 204). Moreover, they express IL-25, a cytokine that increases release of IL-4, -5, and -13 from T cells. Serum IL-25 is increased in patients with active EGPA compared to inactive disease or healthy controls. It is also detectable in eosinophils from lesional biopsies. T cells in these biopsies and in the blood express the IL-25 receptor IL-17RB (205). This suggests a feed-forward loop between eosinophils and Th2 cells in EGPA.

Neuropathy is a common symptom of EGPA (196). Interestingly, different mechanisms lead to nerve damage depending on the presence or absence of MPO–ANCA. In patients with autoantibodies, MPO–ANCA-induced necrotizing vasculitis results in ischemic damage to the nerves (206–209). In the absence of autoantibodies, massive eosinophil infiltration into the epineurium and occasionally endoneurium is observed. These eosinophils are degranulating and cytotoxic to nerves (209, 210). Sometimes eosinophils form part of the inflammatory infiltrate surrounding necrotizing vessels (209, 211, 212). This may accelerate damage of blood vessels because eosinophils were shown to be directly cytotoxic to endothelial cells *in vitro* (34, 213). This damage may be mediated by ECP, which is deposited on endothelial surfaces in patients with eosinophilic endomyocarditis (214–216), or by MBP, which is cytotoxic *in vitro* (34).

Eosinophil chemotaxis into affected tissues in EGPA patients occurs in response to eotaxin-3. Serum levels of eotaxin-3 are substantially higher in EGPA patients with active disease compared to those with inactive disease, healthy controls, or patients with other eosinophil-associated diseases (217, 218). In contrast, there is no increase in serum eotaxin-1 or -2 (217). Eotaxin-3 is also readily detected in biopsies of affected tissues from EGPA patients (217). Eotaxin-3 localizes to endothelial cells of small vessels, smooth muscle cells of small arterioles, the perineurium of the sural nerve, and the respiratory epithelium of the nose. An analysis of single-nucleotide polymorphisms in the eotaxin-3 gene in 161 EGPA patients found no significant associations (218), suggesting that eotaxin-3 polymorphisms may not be causal in EGPA.

The strongest evidence for a pathogenic role of eosinophils in EGPA comes from novel biological treatments that target IL-5 and thereby drastically reduce eosinophil levels. Two open-label trials with the anti-IL-5 antibody mepolizumab demonstrated its efficacy as a steroid-sparing agent and its ability to induce remission over 9 months (219–221). Upon termination of mepolizumab treatment, the majority of patients developed relapses. In one trial, eosinophil count and serum ECP were strongly correlated with disease activity (221). A double-blind randomized placebo-controlled trial of mepolizumab in EGPA is currently ongoing (http://ClinicalTrials.gov identifier: NCT02020889).

Several key findings amount to moderate evidence for a pathogenic role of eosinophils in EGPA. (1) The number of eosinophils and serum ECP correlate with disease severity. (2) Eosinophil infiltration and degranulation in tissues causes organ damage. (3) A potential feed-forward loop between Th2 cells and eosinophils may propagate disease. (4) IL-5-targeted therapies showed beneficial effects.

### Eosinophilic Myocarditis

Myocarditis is the inflammation of the heart muscle with or without damage or necrosis of adjacent myocytes in the absence of an ischemic event (222). A wide range of causes from viral, bacterial, and parasitic infections to toxic effects of drugs or hypersensitivity reactions can cause myocarditis, and in many cases, the etiology is unknown (223). Autoimmune processes often play a role either causally or as postinfection autoimmunity: autoantibodies against cardiac antigens are present in the majority of myocarditis patients, myocarditis is associated with other autoimmune diseases, and some patients benefit from immunosuppressive treatment (224, 225). Animal models provide further evidence for autoimmune mechanisms. Cardiac autoantibodies induce disease in rats, and immunization with cardiac myosin peptide in adjuvants induces experimental autoimmune myocarditis (EAM) in mice (224).

Eosinophils form a major part of the inflammatory infiltrate in subtypes of myocarditis, namely in eosinophilic myocarditis and giant cell myocarditis. These subtypes are usually idiopathic. Eosinophilic myocarditis is associated with hypereosinophilic syndrome (HES) and EGPA, but it can also develop in the absence of eosinophilia. About one-third of EGPA patients and 20–50% of HES patients develop cardiovascular manifestations (196, 197, 226–229). Myocarditis is more frequent in ANCA-negative EGPA patients (196, 229). Parasitic infections and hypersensitivity reactions to drugs are other potential causes of eosinophilic myocarditis (230, 231). Giant cell myocarditis and eosinophilic myocarditis are usually treated with strong immunosuppressive agents.

Eosinophils likely play a pathogenic role in the heart (227, 232, 233). Eosinophil granule proteins are deposited in the myocardium during eosinophilic myocarditis and may be cytotoxic to cardiomyocytes (61, 234–236). Eosinophils have also been proposed to activate cardiac mast cells (237) or release prothrombotic tissue factor (238). In HES, eosinophils are thought to damage the endocardium, which results in thrombosis and endocarditis and eventually leads to endomyocardial fibrosis and valvular complications (226, 228). Animal studies further strengthen the evidence that eosinophils contribute to pathology and mortality in eosinophilic myocarditis. Hypereosinophilic mice with transgenic expression of IL-5 (IL-5Tg) spontaneously develop eosinophilic myocarditis at a low frequency (239). We found that induction of EAM in these IL-5Tg mice reliably induces eosinophilic myocarditis with over 60% of the heart-infiltrating cells being eosinophils (240). Induction of EAM in A/J mice causes myocarditis with numerous infiltrating eosinophils (241). Blockade of IL-4 in this model reduces eosinophil infiltration and disease severity (241). Induction of EAM in BALB/c mice that lack interferon (IFN)γ and IL-17A (IFNγ^−/−^IL-17A^−/−^) results in severe eosinophilic myocarditis with about 50% fatality by day 21 (242). Ablation of eosinophils in these mice improved survival. In another model, natural killer cell depletion resulted in increased eosinophil infiltration in the heart and aggravated myocarditis. In eosinophil-deficient mice, however, natural killer cell depletion did not increase disease severity (243). These results show that eosinophils are pathogenic in myocarditis.

A major burden of myocarditis lies in the sequela inflammatory dilated cardiomyopathy (DCM), which is the major cause of heart failure in patients younger than 40 years and has a poor 5-year survival rate of less than 50% (244, 245). It is not known at what rate eosinophilic myocarditis patients progress to DCM or how this rate compares to other myocarditis subtypes. By using the EAM model, we found that eosinophil-deficient mice are protected from DCM following myocarditis, while hypereosinophilic IL-5Tg mice developed more severe DCM. This process was dependent on eosinophil-derived IL-4 (240). This suggests that eosinophils drive the chronic disease that ensues myocarditis and impair cardiac function.

Little is known about the mediators that induce eosinophil infiltration into the heart. We found increased expression of eotaxin-1 and eotaxin-3 in endomyocardial biopsies from patients with eosinophilic myocarditis compared to chronic lymphocytic myocarditis (24). In the eosinophilic myocarditis mouse model of EAM in IFNγ^−/−^IL-17A^−/−^ mice, cardiac expression of eotaxin-1 and -2 is highly increased compared to naïve mice or WT controls (24, 242). In this model, the eotaxin-CCR3 pathway is necessary for eosinophil trafficking to the heart during myocarditis (24).

In summary, there is substantial evidence that eosinophils play a pathogenic role in myocarditis during acute and chronic stages. Several studies in animal models offered mechanistic insight into how eosinophils contribute to myocarditis. It will be interesting to see if eosinophil-targeted therapies in patients with HES or EGPA will reduce the incidence of eosinophilic myocarditis in this high-risk group.

### Neuromyelitis Optica

Neuromyelitis optica (NMO) is a demyelinating disease of the central nervous system (CNS) that usually affects the optic nerve and spinal cord. Lesions are necrotic, cavitary, and infiltrated with macrophages and granulocytes. NMO is an autoimmune disease. Anti-aquaporin 4 (AQP4) autoantibodies are present in the majority of patients. These pathogenic antibodies are highly specific for NMO and are one of the features that distinguish it from multiple sclerosis. NMO patients often carry multiple other autoantibodies, and there is a strong association with other autoimmune diseases. Moreover, NMO is much more common in women than men (246).

Neuromyelitis optica has only recently been distinguished from multiple sclerosis with eosinophil infiltration being one of the distinctive features (247). The first description of eosinophil infiltration in NMO lesions was by Lucchinetti and colleagues in 2002. In analyzing lesions from NMO patient autopsies, they found eosinophil infiltration in early active lesions (248). Eosinophils infiltration is located meningeal and perivascular in spinal cord lesions. Both intact and degranulating eosinophils are found (248). Since this original observation, multiple studies have described eosinophil infiltration in the spinal cord, optic nerve (249), brainstem (250, 251), and cerebrospinal fluid (252). Another study found that the cerebrospinal fluid from patients with NMO contains higher levels of eotaxin-2, eotaxin-3, and ECP compared to healthy controls or multiple sclerosis patients. In addition, stimulation of cerebrospinal fluid cells with myelin oligodendrocyte glycoprotein results in increased IL-5 production in NMO compared to controls (253). Together, these studies clearly establish that eosinophils infiltrate and degranulate in NMO lesions, which suggest a pathogenic role for eosinophils.

A recent elegant study used *in vitro* experiments and a mouse model to determine the role of eosinophils in NMO (254). Bone marrow-derived eosinophils exhibit ADCC when cocultured with a cell line expressing AQP4 in the presence of anti-AQP4. Similar effects of eosinophils are observed on spinal cord slide cultures. Stimulation of eosinophils with platelet-activating factor (PAF), which induces the release of EPX, results in damage to spinal cord slice cultures independent of anti-AQP4 antibody. The authors developed a mouse model of NMO by continuously infusing anti-AQP4 antibodies and human complement intracerebrally for 3 days. In this model, depletion of neutrophils, eosinophils, or both reduces pathology. Likewise, eosinophil-deficient mice have less severe lesions. Induction of disease in hypereosinophilic mice results in more severe lesions with increased eosinophil and neutrophil infiltration (254). This clearly established a pathogenic role for eosinophils in NMO and highlights mechanisms (ADCC and degranulation) by which eosinophils can damage neural tissues.

### Primary Biliary Cirrhosis

Primary biliary cirrhosis is a chronic disease of the small intrahepatic bile ducts that eventually leads to cirrhosis. It shows several hallmarks of an autoimmune disease: highly specific antimitochondrial autoantibodies, association with other autoimmune diseases, a female to male ratio of 10:1, and a strong genetic component (255). Histologically, damaged biliary epithelial cells and infiltration of the portal area with plasma cells, T cells, NK cells, macrophages, neutrophils, and eosinophils are visible (255, 256). Cytokine expression in the liver of primary biliary cirrhosis patients is similarly mixed. Compared to other liver diseases, increased hepatic expression of IL-5, IL-6, IFNγ, TGFβ, and IL-2 has been noted (257, 258). Recent studies also identified key Th1 and Th17 cytokines in the liver (259) and on blood cells (260) and a decreased T regulatory to Th17 cell balance in peripheral blood cells (261).

Patients with primary biliary cirrhosis have a higher frequency and increased absolute numbers of eosinophils in peripheral blood and the liver, particularly around damaged bile ducts (262–265). Eosinophil infiltration is higher in the early stages of the disease (stages I–II versus III–IV) (263, 265). Increased eosinophil infiltration was positively associated with liver IL-5 expression (258) and mast cell infiltration (264). Infiltrating eosinophils are degranulating, releasing ECP, MBP, and EDN, which can also be detected in the serum (262, 265). Some patients have autoantibodies to EPX (266), although it is unclear whether these have any pathologic relevance. Two of the established mouse models for primary biliary cirrhosis show eosinophil infiltration in the liver and could be useful for further studies on the role of eosinophils (267–269).

Ursodeoxycholic acid (UDCA) is the only approved drug for primary biliary cirrhosis patients. UDCA can delay disease progression and improve liver biochemistry (255). Of note, a higher frequency of blood eosinophils is associated with better response to UDCA treatment (263). UDCA treatment decreases the frequency and number of eosinophils in the blood (263, 270) and the liver (265) and decreases degranulation of tissue eosinophils and serum MBP and EDN (265). UDCA may suppress tissue eosinophilia by altering dendritic cells and the local cytokine milieu (271).

In some cases, eosinophilia may precede the detection of liver pathology, suggesting that eosinophils are involved early in the disease processes. One study reports on four cases of asymptomatic women with eosinophilia detected during random investigation. All of them had elevated liver enzymes and were diagnosed with primary biliary cirrhosis (272). In another patient eventually diagnosed with primary biliary cirrhosis, eosinophilia was detected 18 months prior to diagnosis, but liver enzymes were still normal 12 months prior to diagnosis, suggesting that eosinophilia can precede overt liver pathology (273). In conclusion, there is some evidence for a role of eosinophils in the early stages of primary biliary cirrhosis.

### Other Diseases

Several rare diseases with a possible autoimmune etiology are associated with eosinophils. Usually only case reports or small case series are available for these diseases, making it very difficult to assign a pathologic or protective role to eosinophils.

#### Eosinophilic Cellulitis

Eosinophilic cellulitis (Wells’ syndrome) is a very rare skin disease characterized by recurrent edematous erythema. Eosinophilic cellulitis is potentially associated with EGPA, HES, UC, or other causes, but the etiology is unknown (274, 275). The typical histopathological sign is flame figures, the focal accumulation of disintegrating eosinophils and collagen fibers. Early stages are characterized by predominantly eosinophilic infiltration (274). Blood eosinophilia is present in 15–67% of patients (275). Blood eosinophils from patients with eosinophilic cellulitis express the high-affinity IL-2 receptor CD25 (276). *In vitro*, IL-2 treatment of CD25^+^ eosinophils resulted in priming and increased release of ECP upon subsequent PAF stimulation. This suggests that eosinophils in patients with eosinophilic cellulitis may degranulate more easily. Indeed, extracellular MBP staining is readily observed in flame figures (277, 278) and may be in amyloid form, a sign of large-scale degranulation (279). Eosinophil chemotactic factors CCL17 and CCL24 have been detected in lesions (280). It is possible that eosinophils play a pathogenic role through degranulation.

#### Eosinophilic Fasciitis

Eosinophilic fasciitis is characterized by thickening and inflammation of the fascia resulting in painful swelling and progressive induration of the skin and soft tissues (281, 282). The etiology of eosinophilic fasciitis is unknown. Autoimmune disease, infections, drugs, physical exertion, and other factors are discussed as potential triggers (283). There is no clear predominance by sex. Antinuclear antibodies are present in 15–20% of patients (284). Blood eosinophilia is present in most cases (60–90%), and eosinophils infiltrate the fascia and sometimes the perimysium (285–287). This increase in eosinophils is not always present and may be a transient feature. In one study, blood or tissue eosinophilia was not associated with the clinical outcome (cure versus residual fibrosis) (286).

#### IgG4-Related Disease

The fibroinflammatory IgG4-related disease can affect multiple organs including the pancreas, salivary and lacrimal glands, lungs, retroperitoneum, and other tissues (288, 289). While it seems to be an immune-mediated disease, no target antigen (autoimmune or microbial) has been identified (290). The etiology and triggering factors are unknown (289). High serum IgG4 is present in 60–70% of patients (291), but it is unclear whether these IgG4 antibodies are directly pathogenic (289, 290, 292, 293). Key histopathological features are a dense lymphoplasmacytic infiltrate, storiform fibrosis, and obliterative phlebitis (288). Peripheral blood eosinophilia is found in about 30% of patients (294). Eosinophils also infiltrate the tissues and are present in the majority of lesions. Eosinophil infiltration is usually mild to moderate but can be predominant in some cases (288). To date, there is no clear evidence for or against a pathogenic role of eosinophils. However, several potential mechanisms have been hypothesized: antigen presentation, release of profibrotic factors, and promotion of plasma cell survival for IgG4 production (290).

## Autoimmune Diseases in Patients with Eosinophil-Associated Diseases

Eosinophils are a key feature of asthma, hypereosinophilic syndromes, and eosinophilic gastrointestinal diseases. An increased frequency of autoimmune diseases in patients with these eosinophil-associated diseases would be suggestive of a possible role for eosinophils in autoimmunity.

### Hypereosinophilic Syndrome

There are numerous case reports of patients with hypereosinophilic syndrome who also suffer from an autoimmune disease including ulcerative colitis, autoimmune hepatitis, autoimmune thyroiditis, multiple sclerosis, systemic lupus erythematosus, antiphospholipid syndrome, myasthenia gravis, and rheumatoid arthritis (295–310). Some of these patients had more than one autoimmune disease. From these case reports, the overall frequency of autoimmune diseases in HES cannot be determined. It is also not clear if HES precedes autoimmune disease or *vice versa*. In a trial of mepolizumab therapy for HES, 5 of 78 patients under follow-up developed autoimmune diseases (rheumatoid arthritis, polymyalgia rheumatica, temporal arteritis, lichen planus, and autoimmune thrombocytopenia) (311). These diseases were likely revealed by the tapering of glucocorticoids, but an effect of mepolizumab cannot be excluded. It seems that these are in excess of the expected prevalence of autoimmune diseases; however, future studies are required to determine if this is the case.

### Eosinophilic Gastrointestinal Diseases

Autoimmune diseases may be associated with eosinophilic gastrointestinal diseases. A recent literature review summarized case reports of autoimmune connective tissue diseases (SLE, rheumatoid arthritis, systemic sclerosis, and inflammatory myositis) in patients with eosinophilic gastroenteritis (312). These patients were mostly female even though eosinophilic gastroenteritis shows a male predominance. The issue remains that case reports do not allow for any conclusion of association. A recent population-based cohort study on eosinophilic esophagitis found that the risk of several autoimmune diseases was substantially increased in patients compared to controls (313). Eosinophilic esophagitis patients had an increased risk of celiac disease, Crohn’s disease, ulcerative colitis, rheumatoid arthritis, lupus, systemic sclerosis, Hashimoto’s thyroiditis, and multiple sclerosis. No increased risk was found for pernicious anemia or vitiligo. Whether female compared to male eosinophilic esophagitis patients were more likely to also suffer from an autoimmune disease was not assessed. Genome-wide association studies have identified risk loci for eosinophilic esophagitis that were previously associated with autoimmune diseases, suggesting a potential common genetic cause (314). It will be interesting to see whether eosinophilic esophagitis patients with autoimmune comorbidities differ from those without and whether an association of other eosinophilic gastrointestinal diseases with autoimmune diseases can be proven.

### Asthma

Eosinophils are a prominent feature of allergic asthma and, to a lesser extent, non-allergic asthma (315). A potential role for autoimmune processes in asthma has been proposed (316). Certain autoantibodies are found more frequently in patients with asthma than in healthy controls (317), and some studies have found a positive association of asthma with autoimmune diseases (318–320). However, others report negative associations between these two conditions (321, 322). It has also been suggested that sex hormones may contribute to asthma severity (323). Non-allergic asthma has a female predominance, while allergic asthma does not (324, 325). Whether autoimmunity plays a role in asthma pathology or is increased in affected individuals remains controversial.

To date, there is no strong evidence for increased autoimmune diseases in patients with HES, asthma, or eosinophilic gastrointestinal disease, and only one study determined an increased risk in EoE patients. Future cross-sectional or cohort studies will be required to determine if the prevalence of autoimmune diseases is truly higher in patients with eosinophil-associated diseases.

## Conclusion

There is clear evidence for a pathogenic role of eosinophils in several autoimmune diseases (Table 1). Protective functions of eosinophils have not been identified. Eosinophils contribute to autoimmune diseases in vastly different organs, from the CNS to the skin, gastrointestinal tract, and cardiovascular system. These include tissues where eosinophils reside in healthy individuals, such as the intestine, as well as those where eosinophil are usually absent, such as the heart or CNS. In all of these organs, eotaxins seem to be the main chemokines for eosinophil recruitment. Different eotaxins attract eosinophils to different tissues. Eotaxin-1 is essential for eosinophil trafficking to the intestine, while eotaxin-3 is most important in EGPA. Both eotaxin-1 and -3 attract eosinophils to the heart and eotaxin-2 and -3 recruit eosinophils to the CNS in NMO. Eosinophil infiltration into tissues is usually accompanied by eosinophilia, which may be transient, and is often caused by an increase in serum IL-5 or tissue IL-5. Other cytokines like GM-CSF may also be increased and contribute to eosinophilia. Compared to healthy controls, eosinophils from affected tissues or blood of patients show an activated phenotype, upregulating CD11b and CD69, and releasing cytokines such as IL-25, IL-6, IL-8, and IL-1β.

**Table 1:** **Autoimmune diseases with potential eosinophil involvement**.

Disease	Level of evidence	Potential mechanism	Eosinophil recruitment	Tissue infiltration	Blood eosinophilia
Bullous pemphigoid	Strong	Eosinophil-derived proteases degrade extracellular matrix resulting in dermal–epidermal separation	Eotaxin-1, expressed by keratinocytes	Yes	Yes, likely associated with disease severity
Inflammatory bowel diseases	Strong	Release of eosinophil peroxidase (EPX), major basic protein, IL-22-binding protein; increase in mucosal barrier permeability; potential effects on enteric nerves	Eotaxin-1 (eotaxin-2 and -3), expressed by multiple cell types	Yes, positively correlated with disease severity	
Eosinophilic granulomatosis with polyangiitis	Moderate	Possible direct cytotoxic effects on endothelial cells, nerves, and other organs involved; prothrombotic effects	Eotaxin-3, expressed by various cell types	Yes (diagnostic criterion)	Yes (diagnostic criterion), increased Th2 cytokines
Eosinophilic myocarditis	Moderate	Possible direct cytotoxic effects on myocytes, endocardium; prothrombotic effects; mast cell activation; release of IL-4 promotes chronic disease	Eotaxin-1, -3	Yes (diagnostic criterion)	Not always present
Neuromyelitis optica	Strong	Release of EPX killing astrocytes through antibody-dependent and complement-dependent cell mediated cytotoxicity	Eotaxin-2, -3	Yes, particularly in early lesions	
Primary biliary cirrhosis	Weak	Unknown, potential cytotoxic effects		Yes, particularly in early stages	Yes

**Figure 2:**
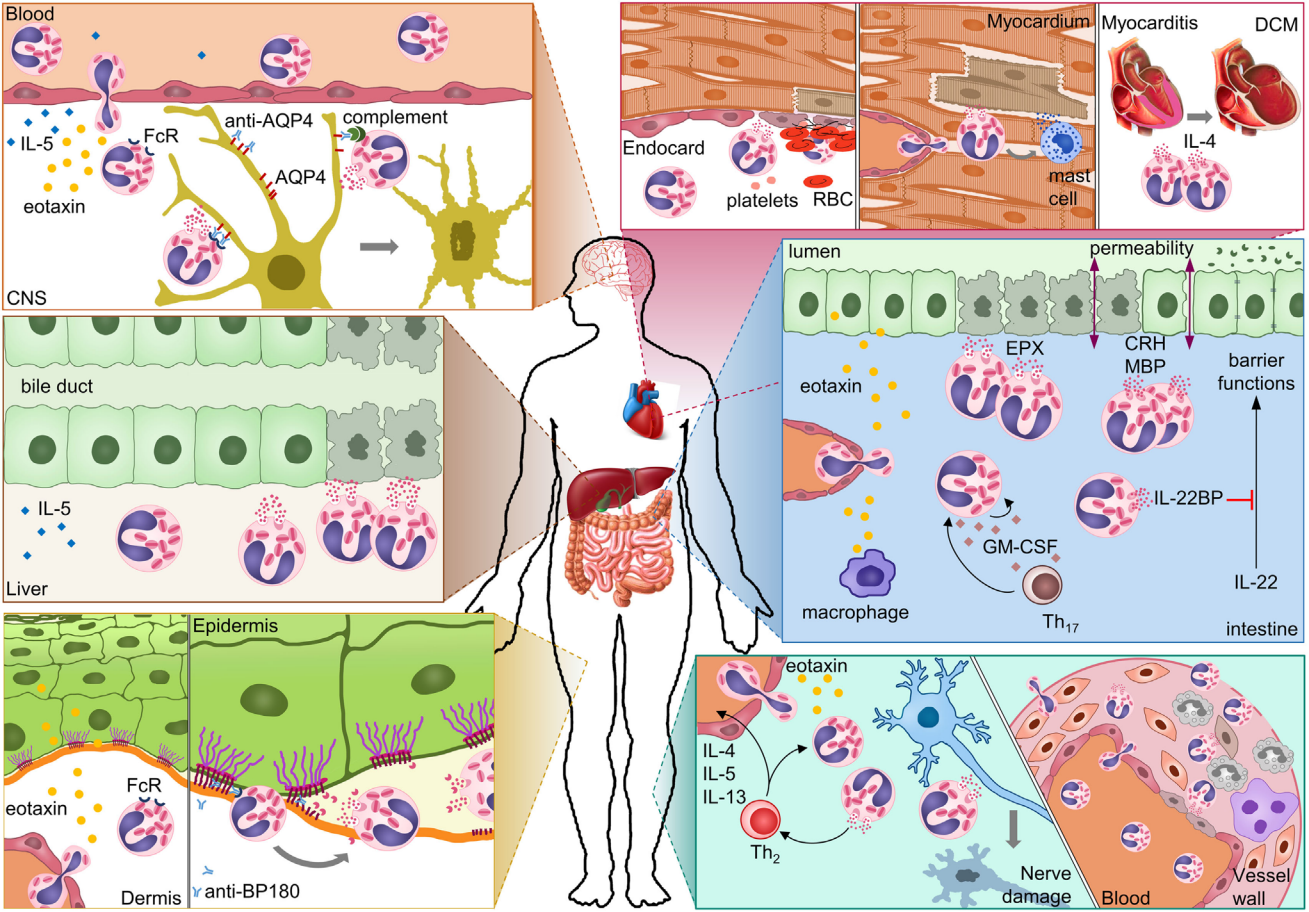
**Possible mechanisms of eosinophil-mediated damage in different autoimmune diseases**. In neuromyelitis optica, eosinophils damage astrocytes through antibody-dependent and complement-dependent cell-mediated cytotoxicity. Eosinophil degranulation in damaged bile ducts was shown for primary biliary cirrhosis. In bullous pemphigoid, eosinophils release proteases that degrade the dermal–epidermal anchoring complex. Eosinophil infiltration in the heart results in damage to the endocardium and myocardium either directly or indirectly through mast cells. Eosinophil-derived IL-4 can drive progression from autoimmune myocarditis to DCM. In inflammatory bowel diseases, eosinophils can damage the mucosa through multiple mechanisms. Eosinophil granule proteins damage epithelial cells and increase epithelial barrier permeability. Eosinophil-derived IL-22BP blocks the protective effects of IL-22 on epithelial cells. GM-CSF may prolong survival and activation of eosinophils in the intestine. In eosinophilic granulomatosis with polyangiitis, eosinophils damage nerves and blood vessels. Abbreviations: CNS, central nervous system; AQP4, aquaporin 4; FcR, Fc receptor; BP180, bullous pemphigoid 180; RBC, red blood cell; DCM, dilated cardiomyopathy; EPX, eosinophil peroxidase; CRH, corticotropin-releasing hormone; MBP, major basic protein; IL-22BP, IL-22-binding protein; GM-CSF, granulocyte-macrophage colony-stimulating factor; Th_17_, T-helper 17 cell; Th_2_, T-helper 2 cell.

Multiple effector mechanisms have been identified (Figure 2). Degranulation of eosinophils is noted most frequently, perhaps because it is easily visualized by immunofluorescence or histology of biopsies. Degranulating eosinophils are often seen adjacent to dying cells, such as endothelial cells of the vasculature and endocardium, nerve cells, dermis, and intestinal mucosa. As a result, direct cytotoxicity of eosinophils to other cells has been proposed as a mechanism in all autoimmune diseases discussed above. EPX, MBP, and ECP all have strong cytotoxic properties, and often multiple mediators are released. The ability of eosinophils to bind antibodies and subsequently degranulate and kill cells links the adaptive autoimmune response to eosinophil effector functions. Antibody-dependent cell-mediated cytotoxicity by eosinophils was shown for NMO. Antibodies in BP likely cause degranulation of eosinophils and blister formation. Eosinophils are frequently associated with tissue remodeling. In BP, eosinophil-derived MMP9 and neutrophil elastase were shown to degrade extracellular matrix proteins resulting in dermal–epidermal separation. In ulcerative colitis, MBP and corticotrophin-releasing factor from eosinophils downregulate tight-junction proteins on epithelial cells, which decrease their barrier function. Eosinophil-derived cytokines modulating the function of other immune or stromal cells also play a role in autoimmune diseases. Eosinophil-derived IL-4 is important for chronic disease progression in myocarditis, and IL-22BP blocks protective functions of IL-22 in UC. In several diseases, eosinophil infiltration was particularly pronounced in the early stages. This may hint to a role in initiation of the autoimmune response, a hypothesis that is difficult to prove in humans.

Particularly for rare diseases, the evidence for eosinophil involvement is mostly based on case reports, which makes it difficult to exclude associations by chance. Because autoimmune disease patients may receive many drugs, it is worth considering that hypersensitivity reactions to drugs are often accompanied by eosinophilia. On the other hand, it is difficult to ascertain the role of eosinophils in patients treated with glucocorticoids, which are highly effective at reducing eosinophil numbers in blood and organs (326, 327). Eosinophils may be reduced to normal or below normal levels in patients under treatment, and this could mask any associations. Novel targeted therapeutics that affect only specific arms of the immune response and do not dampen eosinophils may reveal new associations.

## Future Research Needs

To verify some of the proposed mechanisms and potentially identify new mechanisms of eosinophil-mediated pathology or protection in autoimmune disease, animal models will aid greatly. The lack of *in vitro* or animal models has hampered research in several autoimmune diseases such as EGPA and primary biliary cirrhosis. In addition, epidemiological studies including larger patient cohorts will be required to determine whether autoimmune diseases are indeed increased in patients with eosinophil-associated diseases such as eosinophilic esophagitis, hypereosinophilic syndrome, or asthma. Finally, viewing and analyzing autoimmune diseases with eosinophil involvement as a group with possible shared mechanisms may advance our understanding and point to common processes.

## Author Contributions

ND reviewed the literature and wrote the manuscript. NR conceptualized the idea for the review and edited the manuscript. DČ edited the manuscript and contributed to defining the scope and content of the review.

## Conflict of Interest Statement

The authors declare that the research was conducted in the absence of any commercial or financial relationships that could be construed as a potential conflict of interest.
